# Multi-Orbital Charge Transfer into Nonplanar Cycloarenes Revealed with CO-Functionalized STM Tips

**DOI:** 10.1021/acs.jpclett.5c03268

**Published:** 2026-01-21

**Authors:** Anja Haags, Alexander Reichmann, Zilin Ruan, Qitang Fan, Larissa Egger, Hans Kirschner, Tim Naumann, Simon Werner, Olaf Kleykamp, Jose Martinez Castro, Felix Lüpke, François C. Bocquet, Christian Kumpf, Serguei Soubatch, Alexander Gottwald, Georg Koller, Michael G. Ramsey, Mathias Richter, Jörg Sundermeyer, Peter Puschnig, J. Michael Gottfried, F. Stefan Tautz, Sabine Wenzel

**Affiliations:** † Peter Grünberg Institut (PGI-3), 163337 Forschungszentrum Jülich, 52425 Jülich, Germany; ‡ Jülich Aachen Research Alliance (JARA), 27267 Fundamentals of Future Information Technology, 52425 Jülich, Germany; ¶ Experimental Physics IV A, 9377 RWTH Aachen University, 52074 Aachen, Germany; § Institute of Physics, 12652 University of Graz, NAWI Graz, 8010 Graz, Austria; ∥ Department of Chemistry, Marburg University, 35043 Marburg, Germany; ⊥ Physikalisch-Technische Bundesanstalt (PTB), 10587 Berlin, Germany; # Experimental Physics II B, 9377 RWTH Aachen University, 52074 Aachen, Germany; ∇ II. Physikalisches Institut, Universität zu Köln, Zülpicher Straße 77, 50937 Köln, Germany

## Abstract

On-surface synthesis enables the tunable preparation of diverse molecular systems with tailored properties. Recently, the highly selective synthesis of kekulene (>99%) on Cu(111) and isokekulene (92%) on Cu(110) from the same molecular precursor was demonstrated. Scanning tunneling microscopy (STM) with CO-functionalized tips can identify individual molecules based on their geometric structure at low coverage on Cu(110) but also reveals complex features arising from electronic contributions near the Fermi energy. Here, we investigate the origin of these features by simulating STM images based on a weighted sum of multiple molecular orbitals, for which we employ weights based on the calculated molecular-orbital projected density of states. This analysis provides direct experimental evidence for charge transfer from the surface into multiple formerly unoccupied molecular orbitals for single molecules of kekulene as well as isokekulene in its two nonplanar adsorption configurations. In comparison, the area-integrating photoemission orbital tomography technique confirms the charge transfer as well as the high selectivity for the formation of a full monolayer of predominantly isokekulene on Cu(110). Our STM-based approach is applicable to a wide range of adsorbed molecular systems and specifically also suited for strongly interacting surfaces, nonplanar molecules, and compounds accessible only in extremely low yields.

## Graphical abstract

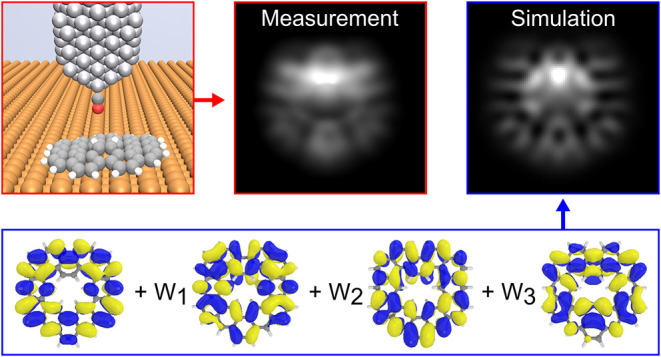

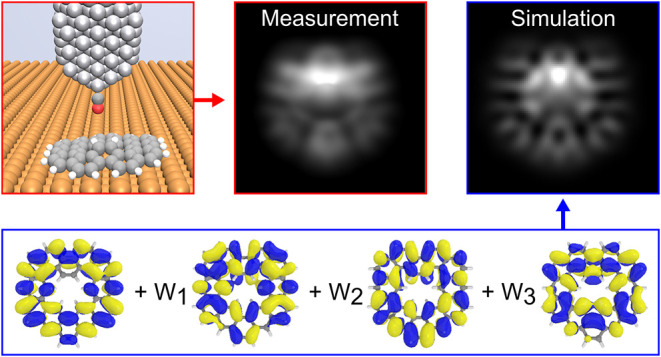

Molecular systems have a wide variety and range of tunable properties, and therefore great potential for applications in electronics,^1^ as molecular switches,^2^ sensors,^3^ solar cell materials,^4^ and sustainable single-atom catalysts.^5^ A diverse class of carbon-based molecules with variable geometric, electronic, optoelectronic, and magnetic properties have been realized in recent years by on-surface synthesis.^6–12^ The outcome of on-surface synthesis reactions can not only be steered by the choice of specialized molecular precursors prepared in solution but also by employing different surface compositions and structures.^13–17^ Most recently, some of the authors of the present study have reported the highly selective synthesis of kekulene on Cu(111) and its nonplanar isomer isokekulene on Cu(110) by different intramolecular dehydrogenation reactions within the same precursor molecule.^18^ Single isokekulene molecules on Cu(110) were identified by constant-height scanning tunneling microscopy (STM) using functionalized tips, which is an established technique for imaging the geometric structure of molecules.^19–22^ However, in that work, the images showed additional complex features due to electronic contributions close to the Fermi energy, which was suggested to be a sign of a strong molecule–surface interaction.^18^ The origin of these features has yet to be investigated in detail.

The electronic structure of adsorbed molecules can be studied with established methods such as angle-resolved photoemission spectroscopy (ARPES), especially in combination with density functional theory (DFT) in the form of photoemission orbital tomography (POT).^23–32^ Recently, POT has been used to study the aromaticity of kekulene synthesized on Cu(111).^9^ Although this technique has so far mostly been applied to planar molecules,^23^ there has been recent progress in using it to determine the adsorption geometry of nonplanar molecules^33,34^ as well as to identify nonplanar adsorption geometries of planar molecules.^35,36^ However, area-integrating methods such as ARPES and POT can only be applied on ordered molecular layers at a significant surface coverage. In contrast, on-surface synthesis, especially when performed by tip manipulation, often leads to low coverages or even single molecules of the desired product, and various byproducts or unreacted precursor molecules might be present on the same surface.^11,12^

Scanning tunneling spectroscopy (STS) is well-suited to detect electronic states of single adsorbed molecules. By tuning the bias voltage in the STM to the energetic positions of specific orbitals, images of the real space distribution of the highest occupied and lowest unoccupied molecular orbitals can be recorded.^37,38^ Other orbitals can be isolated by recording differential conductance (d*I*/d*V*) images at their specific energies.^39–41^ For nonplanar molecules, the agreement with the theoretical local density of states can be improved by conducting the measurements at constant d*I*/d*V* instead of constant current or height.^42^ The resolution of both STS spectra and d*I*/d*V* images can also be improved by tip functionalization.^41^ Specifically, functionalization with CO allows for an additional tunneling channel into *p*-wave orbitals of the tip, which leads to imaging that can be related to the lateral derivative of the local density of electronic states.^43^ However, even with functionalized tips, specific molecular orbitals might lie too close in energy to distinguish them in single d*I*/d*V* images. Recently, the deconvolution of orbitals as close as 50 meV in energy was achieved by employing a detailed STS analysis,^41^ the so-called feature detection algorithm.^44^ However, this approach is based on recording a complete d*I*/d*V* spectrum at every pixel of an image of the molecule, which is highly time-intensive and requires excellent stability of the sample and the microscope. These requirements are particularly challenging for nonplanar molecules, and the method is limited in resolving exactly degenerate orbitals or those that are strongly broadened through hybridization with substrate states.

Here, we present an alternative approach to probing the occupation of multiple orbitals at a specific energy for nonplanar, strongly interacting molecules using only single constant-height STM images recorded with a CO-functionalized tip. In an analogous fashion to POT, the measured STM images are compared to simulations constructed as the weighted sum of theoretical orbitals with the weights determined based on the molecular-orbital projected density of states (MOPDOS) calculated from DFT. The results of this analysis are evaluated in comparison to the results of POT on a full monolayer of the same molecule.

Isokekulene and small amounts of kekulene on Cu(110) are prepared by a combined in-solution and on-surface approach, as reported previously.^18^ Specifically, the precursor 1,4,7­(2,7)-triphenanthrenacyclononaphane-2,5,8-triene is prepared in solution (details see ref 9) and vapor-deposited onto the surface at 300 K. Upon annealing to 500 K, cyclodehydrogenation yields the respective products. Figure 1a,b shows overview STM images of the resulting isokekulene molecules (typical examples are marked with cyan and dark blue squares) and small amounts of kekulene molecules (a representative example is marked with a green square) at two different coverages on Cu(110). STM images of single molecules of each species are depicted in Figure 1c–e along with the respective chemical structures. Isokekulene is nonplanar and adsorbs in two different configurations, denoted up and down, depending on whether the central benzene ring in the molecule’s pore points upward from or downward toward the surface.

**Figure 1:**
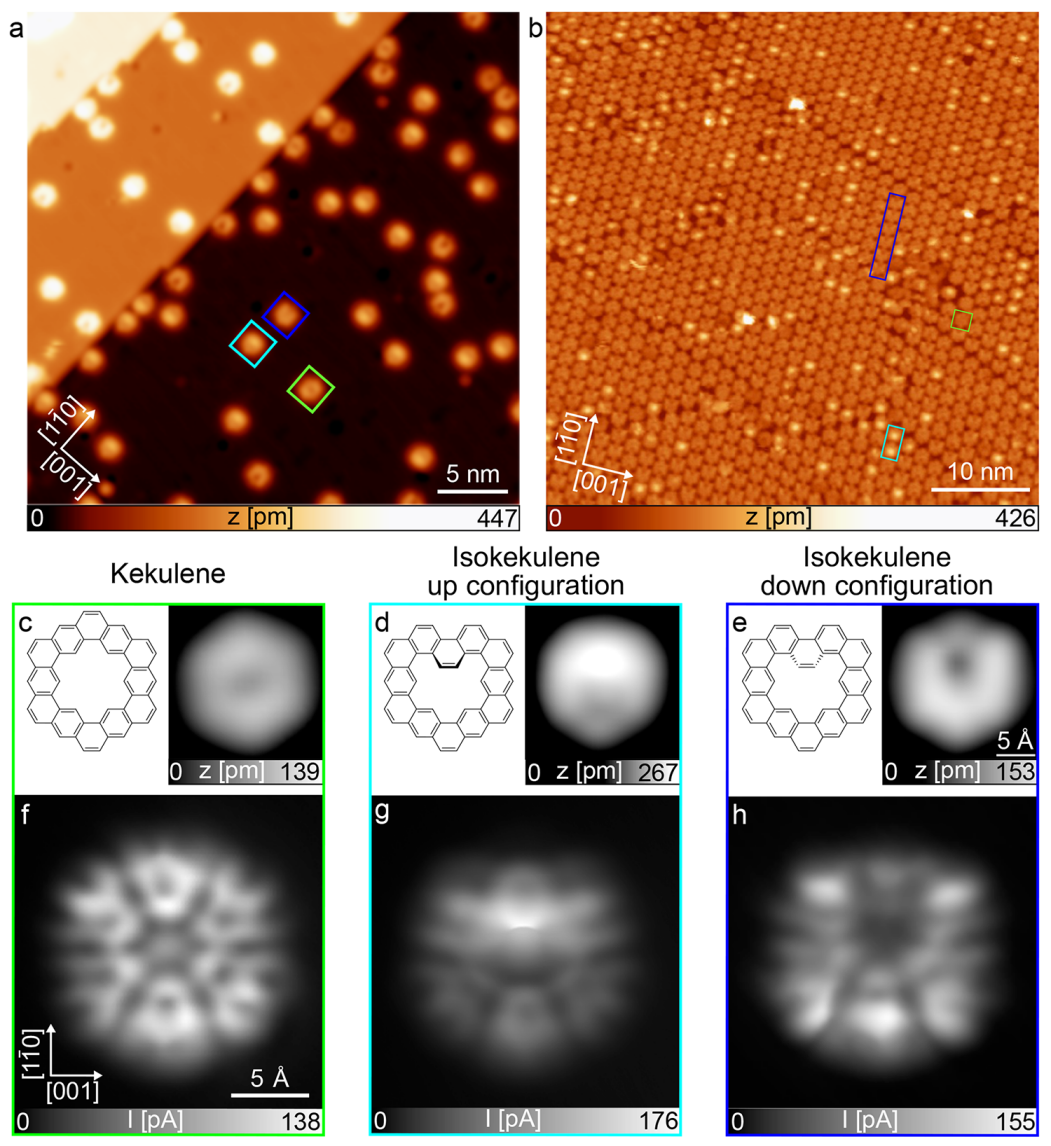
(a) Overview STM image of a low coverage of mainly isokekulene and small amounts of kekulene on Cu(110) recorded on the same surface area as Figure 2a in ref 18 but with a different, metallic tip and improved length calibration. (b) Overview STM image of a full monolayer of the same molecules on Cu(110). (c–e) Small-scale STM images of the surface in (a) showing single molecules of (c) kekulene and isokekulene (d) in the up configuration as well as (e) in the down configuration compared to their respective chemical structures. The STM images were recorded in constant-current mode with (a) *U* = 50 mV and *I* = 20 pA, (b) *U* = 0.76 V and *I* = 0.18 nA, (c) *U* = −1 V and *I* = 150 pA, and (d,e) *U* = 90 mV and *I* = 50 pA. (f–h) Constant-height STM images recorded with a CO-functionalized tip after stabilization at (f, h) *U* = 5 mV or (g) *U* = 20 mV and *I* = 20 pA above the copper substrate and subsequently increasing the height of the tip by (f) *z* = 120 pm, (g) 130 pm, and (h) 125 pm. The images are taken at (a,c–h) 4 K or (b) 100 K surface temperature.

For a more detailed investigation of the density of states around the Fermi energy, the three species were also imaged with a CO-functionalized tip at small bias voltages in constant-height mode as shown in Figure 1f–h. These images are in rough qualitative agreement with the images presented in Figure 2i–k of ref 18, but, here, they were acquired at larger tip–sample distances (on the order of tens of picometers higher), thereby reducing bond-resolved contributions and minimizing the influence of molecular geometry on the STM contrast. This suppression is not fully achieved for isokekulene in the up configuration (see Figure 1g), for which the up-facing central benzene ring still forms a small sharp edge in the contrast, which is indicative of the bond-resolved imaging. At even greater tip–sample distances, however, the remainder of the molecule became barely discernible. For all three species, the persistence of STM contrast beyond purely bond-resolved features at such low bias voltages indicates the presence of electronic states close to the Fermi energy.

To investigate these electronic states in more detail, we first employed angle-resolved photoemission spectroscopy, an established area-integrating technique complementary to the local STM probe. As a reference, Figure 2a shows an ARPES band map of a monolayer of kekulene on Cu(111) measured along the [11̅0]-direction. No molecular emissions are observed close to the Fermi energy, in agreement with the data reported in Figure 3a of ref 9 for the same surface, where only weak molecule–surface interaction was found. In contrast, the band map in Figure 2b, measured along the [11̅0]-direction of Cu(110) for a monolayer dominated by isokekulene (see Figure 1b), reveals pronounced molecular emissions just below the Fermi level.

**Figure 2:**
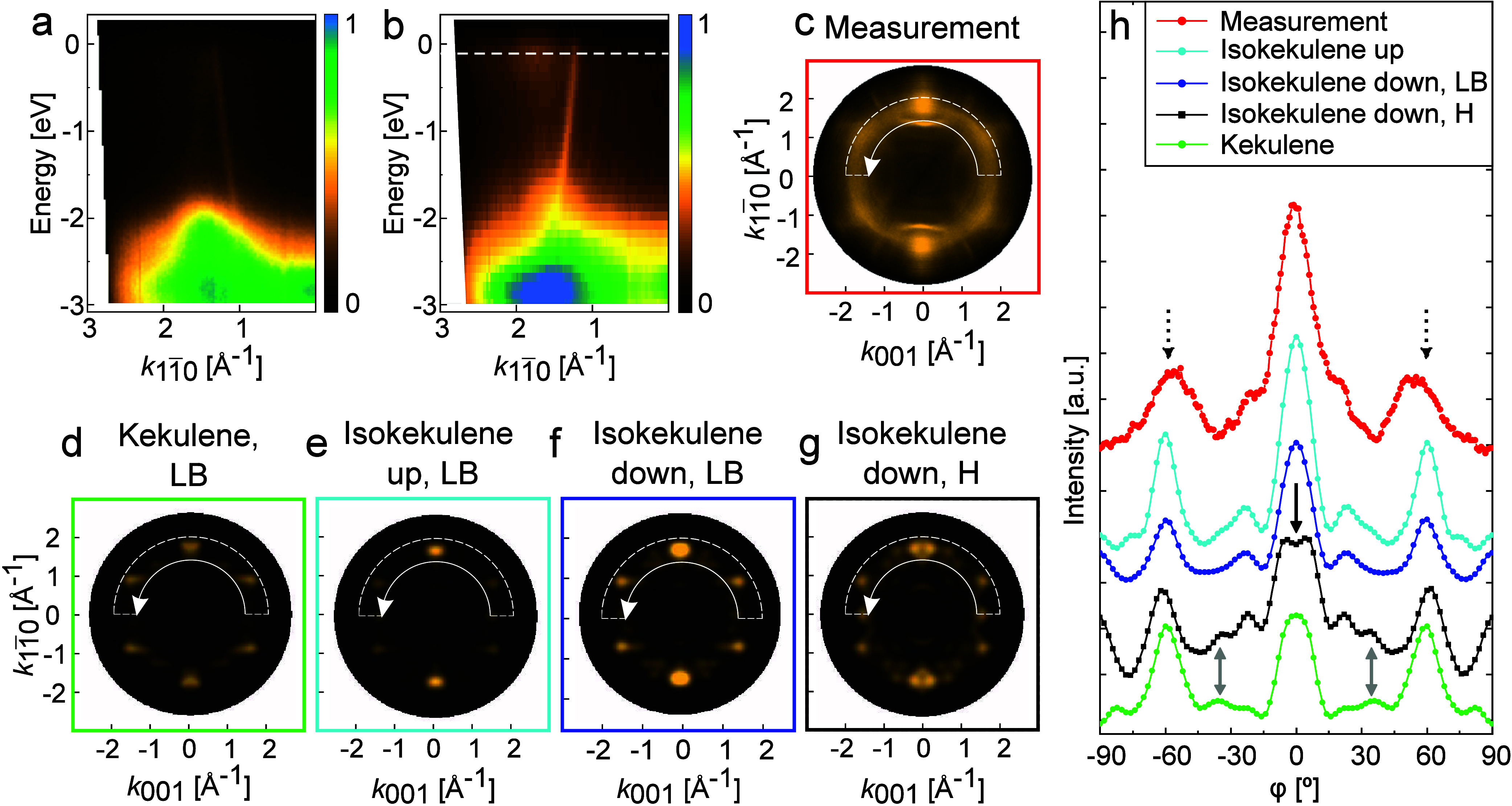
(a) Band map measured along the [11̅0]-direction of Cu(111) in a 3 eV energy range below the Fermi energy on a monolayer of kekulene and (b) band map measured along the [11̅0]-direction of Cu(110) on a monolayer of (iso)­kekulene prepared in the same manner as the surface shown in Figure 1b. (c) *k* map measured at 0.11 eV below the Fermi energy (at the white dashed line in (b)) and the corresponding simulated *k* maps for (d) kekulene, (e) isokekulene in the up configuration, and (f) isokekulene in the down configuration, all at the long bridge (LB) adsorption site, as well as (g) isokekulene in the down configuration at the hollow (H) adsorption site. Note that the theoretical *k* maps include contributions of symmetry-equivalent domains that are indistinguishable by the area-integrating POT. (h) Intensity profiles extracted from the white half circles in the *k* maps. For clarity, the profiles for the different species are shifted along the intensity axis. The measurements were conducted at the Metrology Light Source insertion device^45^ beamline of the Physikalisch-Technische Bundesanstalt (PTB, Germany) (see the Supporting Information for details).

To identify the origin of these emissions, we have calculated the electronic structure of kekulene and isokekulene on copper using DFT. These calculations are based on the relaxed adsorption geometries of the three species reported previously^9,18^ and visualized here in Figure 3a–e. The lower calculated adsorption height of kekulene on Cu(110)^18^ compared to Cu(111)^9^ already suggests a stronger molecule-metal interaction on the more open (110) surface. Additionally, comparison of the energies reported previously^18^ suggests that kekulene and isokekulene in the up configuration preferentially occupy the long bridge site on Cu(110) (see Figure 3b,c), whereas the hollow site is predicted to be energetically favorable for isokekulene in the down configuration (see Figure 3e). This prediction, however, is in conflict with experimental observations since bond-resolved STM suggests that all three species likely adsorb on the long bridge site on the low-coverage surface.^18^ The adsorption site in the full monolayer will be discussed below.

**Figure 3:**
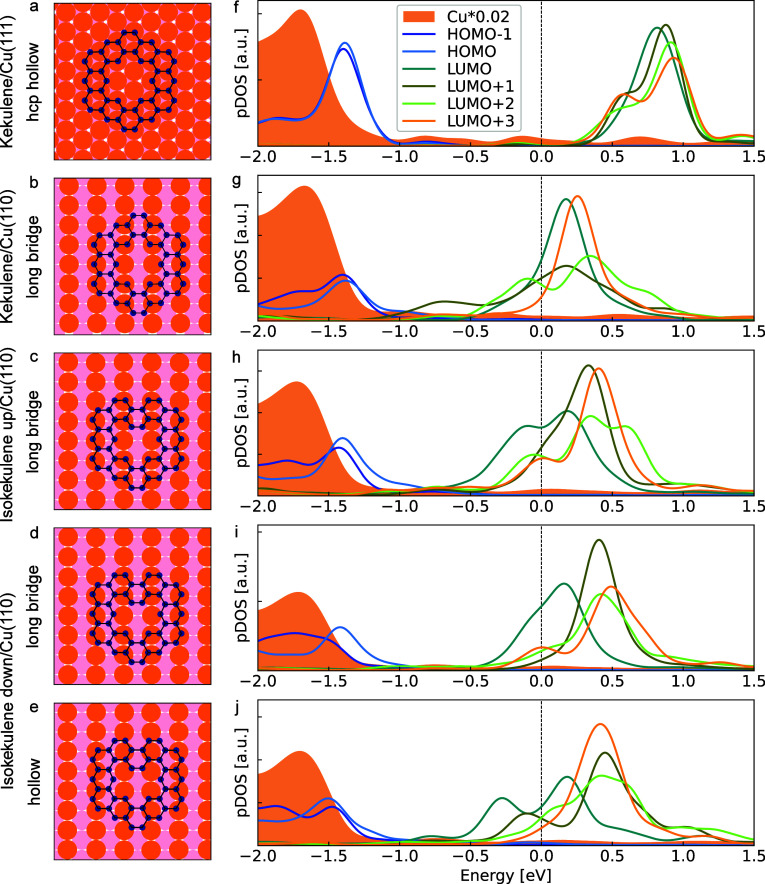
(a–e) Visualization of the adsorption of kekulene (a) at the hcp hollow site of Cu(111) and (b) the long bridge site of Cu(110), as well as isokekulene (c) in the up configuration at the long bridge site on Cu(110) and isokekulene in the down configuration (d) at the long bridge site as well as (e) the hollow site on Cu(110). Copper atoms are orange (top layer) and light pink (second layer), whereas carbon atoms are dark blue. (f–j) Molecular orbital projected density of states (MOPDOS) for HOMO–1 to LUMO+3 of the corresponding molecular layers including the density of states of copper (the latter scaled by a factor of 0.02). Energies are given relative to the Fermi energy. A Gaussian broadening of 0.1 eV was used.

For all adsorption geometries visualized in the left column of Figure 3, the respective MOPDOS was calculated by projecting the wave function onto the molecular gas-phase orbitals (see ref 46 and the Supporting Information for details) and is depicted in the right column of the figure. Figure 3f,g shows the results for kekulene, comparing the adsorption on the weakly interacting Cu(111) surface to the adsorption on Cu(110). For interpreting the MOPDOS of the adsorbed molecules, it is worth noting that the gas-phase kekulene molecule belongs to the D_6*h*_ symmetry group and the orbitals HOMO/HOMO–1 (with E_1*g*_ symmetry), LUMO/LUMO+1 (with E_2*u*_ symmetry), as well as LUMO+2/LUMO+3 (with E_1*g*_ symmetry) are degenerate, respectively. Additionally, the two unoccupied pairs of orbitals are energetically separated by only about 0.1 eV. As can be seen in Figure 3f, these (near) degeneracies as well as the HOMO–LUMO gap of 2.4 eV for the gas-phase molecule remain almost unaffected upon adsorption of a layer of kekulene on Cu(111). Although adsorption reduces the molecular symmetry to C_3*v*_, in which these degeneracies could in principle be lifted, no significant splitting is observed. In contrast, adsorption on Cu(110) induces significant changes of both the energy level alignment and the hybridization between molecular and substrate states (Figure 3g). Several formerly unoccupied orbitals, ranging from LUMO to LUMO+3, become partially filled, indicating charge transfer from the surface to the molecule. The broadening of the corresponding MOPDOS features further reveals stronger hybridization of LUMO+1 and LUMO+2 compared to LUMO and LUMO+3. A similar partial charge transfer and hybridization as for kekulene on Cu(110) is also found for isokekulene on Cu(110) in the up configuration (Figure 3h), as well as the down configuration at both adsorption sites which were considered (Figure 3i,j). Additionally, the MOPDOS curves for the former HOMO and HOMO–1 significantly broaden and overall decrease upon adsorption on Cu(110). This is a result of the strong hybridization with the substrate which generally also leads to charge backdonation from occupied states of the molecule to the metal surface. We note, however, that it is principally not possible to deduce a quantitative amount of total charge transfer from the MOPDOS alone.

For an experimental confirmation of the predicted charge transfer into multiple molecular orbitals, we additionally applied the ARPES-based POT technique. For this, we measured the photoemission angular distribution at 0.11 eV binding energy (marked with the dashed white line in Figure 2b) and plotted the corresponding *k* map (or momentum map, calculated from the angular distribution as described in ref 47) in Figure 2c. The pattern shows two bright emissions at (0.0, ±1.9) Å^–1^ and two pairs of elongated emission lobes at (±1.6, ±1.0) Å^–1^, which suggests a good orientational order of the organic film. Yet, a diffuse, ring-like intensity at *k*_∥_ ≈ 1.6 Å^–1^ points to a certain degree of disorder.

We compared this experimental *k* map with *k* maps simulated for the relaxed geometries and predicted MOPDOS (Figure 3) of kekulene and isokekulene adsorbed on Cu(110), respectively. Using a damped plane wave as final state and following the approach described in ref 46, we computed the *k* maps depicted in Figure 2d–g. For kekulene and isokekulene in the up configuration, we used the long bridge site as the configuration found to be most stable in DFT. For isokekulene in the down configuration, both the long bridge and the hollow sites were considered.

For a detailed comparison, intensity profiles were extracted from all *k* maps by integrating the intensities in the half circles marked with the thin white lines in Figure 2c–g over a radial range from 1.4 Å^–1^ to 2.0 Å^–1^ and plotting the result as a function of the angle (Figure 2h). First, the profile for isokekulene in the down configuration at the hollow site (black curve) has a dip at φ = 0 ° (black solid arrow) and additional small peaks around φ = ±35° (gray arrows), which is inconsistent with the experimental profile (red curve). Thus, it can be excluded that the full monolayer consists of isokekulene in the down configuration sitting at the hollow site. For kekulene (green curve) the intensities of the center peak at φ = 0° and the peaks at around φ = ±60° (indicated by the dotted arrows and present in all profiles) reach similar intensities, again in disagreement with the experiment. In contrast, the profiles of isokekulene at the long bridge site are qualitatively the same for the up and the down configurations (cyan and dark blue curves) in that both show a smaller intensity at φ = ±60° compared to φ = 0°, in best agreement with the experimental intensity profile. This indicates that the monolayer consists predominantly of isokekulene adsorbed at the long bridge site, consistent with the earlier STM findings for isolated molecules.^18^

Additionally, we can conclude that the area-integrating POT allows for a clear distinction between the planar kekulene and its nonplanar isomer, which at the macroscopic scale once again confirms the selectivity toward isokekulene that was found at the molecular scale by STM.^18^ Generally, the good agreement between the measured and the simulated *k*-maps for isokekulene in the long bridge site confirms the charge transfer from the surface into multiple formerly unoccupied molecular orbitals as predicted by the calculated MOPDOS.

In the following, we expand the above approach for comparing the measured to the calculated electronic structure from the momentum space to the real space and from a full monolayer to single molecules. For this, we construct simulated STM images based on the theoretical geometries as well as the predicted MOPDOS (Figure 3) and compare them to the measured STM images (Figure 4a–c). Both *s*-wave and *p*-wave contributions were considered in the simulation. While *s*-wave simulations are calculated from the DFT-calculated wave function according to the Tershoff-Haman approach,^48^ *p*-wave simulations are based on the lateral gradient of the wave function following the description in ref 43. In both cases we employ a three-dimensional (3D) Gaussian broadening to this data similar to the approach presented in ref 49 before taking the modulus squared and a cut perpendicular to the *z* direction to generate the constant-height STM simulation (see Methods Section in the SI for details). A sum over the *p*-wave and *s*-wave contributions in a ratio of 3:1 gave the best agreement with the measurements in our case (see the example of simulations of kekulene calculated with varying ratios in Figure S1). To obtain the wave functions, we employ the gas-phase orbitals of the molecules in the relaxed geometries acquired on Cu(110). The resulting images of individual orbitals are shown in Figure S2 in the Supporting Information. As none of these reproduce the measured images in Figure 4a–c, multiple orbitals must contribute simultaneously. Accordingly, the simulated images in Figure 4d–f were constructed as weighted sums over contributions resulting from the relevant orbitals, with weights taken from the calculated MOPDOS (Figure 3). As different STM images taken in a bias voltage range of ±50 mV appear qualitatively similar (data not shown), we approximated the weights of the orbitals by the ones at the Fermi energy for all species. These simulated images reproduce the general appearance of the experimental images, which confirms the presence of electron density in the formerly unoccupied orbitals around the Fermi energy.

**Figure 4:**
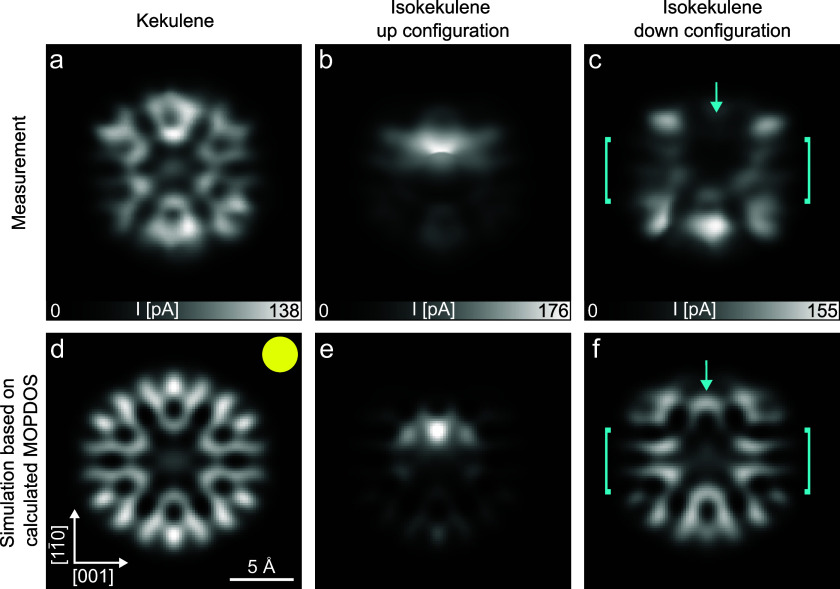
STM images of kekulene (left), isokekulene in the up configuration (middle), and isokekulene in the down configuration (right) on Cu(110), recorded in constant-height mode with a CO-functionalized tip (top row), compared to simulated STM images (bottom row). The simulations employ a *p*-tip to *s*-tip ratio of 3:1 and are based on a weighted sum of the molecular orbitals LUMO, LUMO+1, LUMO+2, and LUMO+3 in the ratio (d) 36:25:21:14, (e) 33:23:19:18, and (f) 46:8:14:17 (based on the calculated MOPDOS). For all three species the relaxed geometry of the molecules at the long bridge site of Cu(110) has been used. The yellow circle visualizes the size of a 3D Gaussian broadening with *FWHM* = 2.8 Å applied to the wave function and its lateral derivative, respectively. Cyan arrows and brackets mark specific areas for which deviations between measurement and simulation are discussed in the text. The STM images were recorded at 4 K surface temperature after stabilization at (a,c) *U* = 5 mV or (b) *U* = 20 mV and *I* = 20 pA above the copper substrate and subsequently increasing the height of the tip by (a) *z* = 120 pm, (b) 130 pm, and (c) 125 pm.

Owing to the flexibility of the presented approach, manual adjustments of the orbital weights can be used to test for possible deviations between the theoretically predicted and the experimental orbital occupations, as exemplified in the Supporting Information. Such deviations appear to be minor in the case of kekulene and isokekulene in the up configuration, however. For isokekulene in the down configuration more significant deviations between the measured and simulated STM images are visible. Specifically, the measured image is darker in the middle (between the cyan brackets overlaid in the image) compared to the brighter areas at the top and the bottom of the molecule with the darkest area, which begins in the pore, extending upward (as marked with the cyan arrow). These features are not reproduced by the simulation, which shows a more even contrast throughout. The agreement could only be improved slightly by removing the LUMO+2 from the weighted sum (see Figure S4i and the Supporting Information). This suggests that the remaining disagreement does not stem from the shape of the orbitals itself, but rather from the relaxed DFT-calculated geometry that these orbitals are placed upon.

Note that the simulated STM image for isokekulene in the down configuration was calculated using the geometry at the long bridge site, which was previously identified in STM,^18^ although DFT predicts that the hollow site is most stable. Figure S3 shows a comparison between simulated STM images employing the geometry at the two different sites in combination with the different weights determined from the calculated MOPDOS at those sites, with and without manual adjustment. Comparing simulations using the same MOPDOS (within one column of Figure S3) or the same geometry (within one row) shows that the results are more sensitive to the choice of orbitals than to the adsorption geometry. However, the agreement with the experimental image cannot be improved by choosing either the MOPDOS (see Figure S3d), the geometry (see Figure S3f), or both (see Figure S3h) from the hollow site instead of the long bridge site for the simulation. Specifically, in the experimental geometry the molecule appears more bent than in all simulated images. The calculated geometries^18^ predict a height difference between the highest and the lowest carbon atoms of ∼1 Å at the long bride and ∼1.2 Å at the hollow sites. This distortion is thus likely underestimated compared to the actual geometry of isokekulene in the down configuration on Cu(110). A similar discrepancy was previously found for PTCDA on MgO using POT.^35^ While POT does not reveal the mismatch in the present case, and generally cannot distinguish the up and the down configuration of isokekulene, the detailed comparison of measured and simulated STM images does uncover a discrepancy between theory and experiment specific to only the down configuration.

In conclusion, we have found evidence for significant charge transfer from Cu(110) into multiple formerly unoccupied molecular orbitals of kekulene and isokekulene by comparing single constant-height STM images to simulations based on DFT. In this approach, theoretical gas-phase orbitals are placed onto relaxed adsorption geometries and their contributions are weighted according to the calculated MOPDOS. Satisfactory agreement is achieved for kekulene and for isokekulene in the up configuration, while a persistent discrepancy for isokekulene in the down configuration suggests that DFT does not fully capture the adsorption geometry and site of this species. In contrast, the area-integrating POT technique cannot distinguish between the up and the down configuration of isokekulene, but can identify the long bridge adsorption site in the full monolayer in agreement with previous STM results for single molecules.^18^ It also indicates charge transfer from the metal into multiple formerly unoccupied molecular orbitals, in full accord with the STM results. Overall, the combination of experimental and theoretical methods presented here provides a versatile framework for elucidating the geometric and electronic structure of both planar and nonplanar molecules on weakly and strongly interacting surfaces.

## Supplementary Material





## Notes

Figures available on simulated STM images employing varying ratios of *s*- and *p*-wave contributions, using single orbitals as well as using different combinations of the geometry and MOPDOS of isokekulene in the down configuration at two different adsorption sites. Supporting Information available on the discussion of manually adjusted orbital weights and on detailed materials and methods (PDF)
Transparent Peer Review report available (PDF)

## @

Chair of Physical Metallurgy, University of Leoben, 8700 Leoben, Austria

## △

Hefei National Research Center for Physical Sciences at the Micro scale, Synergetic Innovation Center of Quantum Information & Quantum Physics, and New Cornerstone Science Laboratory, University of Science and Technology of China, Hefei, Anhui 230026, China.
