# Supplementary material for: Multi-Orbital Charge Transfer into Nonplanar Cycloarenes Revealed with CO-Functionalized STM Tips
Source: J Phys Chem Lett. 2026 Jan 21;17(5):1296–304. doi: 10.1021/acs.jpclett.5c03268 (PMC12884524; doi:10.1021/acs.jpclett.5c03268)
Supplement: Supplementary file 1 [file jz5c03268_si_001.pdf]

Supporting Information

to

Multi-Orbital Charge Transfer into Nonplanar Cycloarenes Revealed with CO-Functionalized STM Tips

Anja Haags,[†,‡,¶] Alexander Reichmann,[§,△] Zilin Ruan,[‖] Qitang Fan,[‖,∇] Larissa Egger,[§] Hans Kirschner,[⊥] Tim Naumann,[‖] Simon Werner,[‖] Olaf Kleykamp,[‖] Jose Martinez Castro,[†,‡,#] Felix Lüpke,[†,‡,@] François C. Bocquet,[†,‡] Christian Kumpf,[†,‡,¶] Serguei Soubatch,[†,‡] Alexander Gottwald,[⊥] Georg Koller,[§] Michael G. Ramsey,[§] Mathias Richter,[⊥] Jörg Sundermeyer,[‖] Peter Puschnig,[§] J. Michael Gottfried,[‖] F. Stefan Tautz,[†,‡,¶] and Sabine Wenzel[*,†,‡,‖]

†*Peter Grünberg Institut (PGI-3), Forschungszentrum Jülich, 52425 Jülich, Germany*
‡*Jülich Aachen Research Alliance (JARA), Fundamentals of Future Information Technology, 52425 Jülich, Germany*
¶*Experimental Physics IV A, RWTH Aachen University, 52074 Aachen, Germany*
§*Institute of Physics, University of Graz, NAWI Graz, 8010 Graz, Austria*
‖*Department of Chemistry, Marburg University, 35037 Marburg, Germany*
⊥*Physikalisch-Technische Bundesanstalt (PTB), 10587 Berlin, Germany*
#*Experimental Physics II B, RWTH Aachen University, 52074 Aachen, Germany*
@*II. Physikalisches Institut, Universität zu Köln, 50937 Köln, Germany*
△*Current Address: Chair of Physical Metallurgy, University of Leoben, 8700 Leoben, Austria*
∇*Current Address: Hefei National Research Center for Physical Sciences at the Micro scale, Synergetic Innovation Center of Quantum Information & Quantum Physics, and New Cornerstone Science Laboratory, University of Science and Technology of China, Hefei, Anhui 230026, China*

E-mail: sabine.wenzel@uni-marburg.de

S1. Supporting Figures

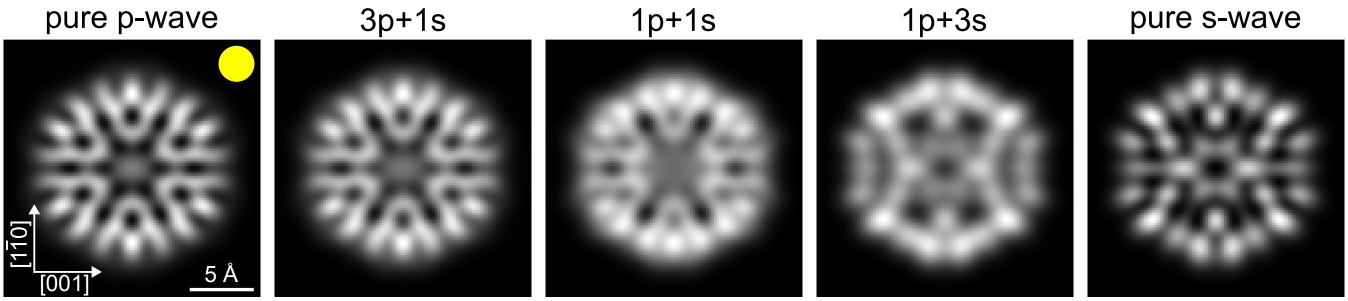

Figure S1: Simulated STM images based on the calculated geometry of kekulene at the long bridge (LB) site on Cu(110) employing a weighted sum of the molecular orbitals LUMO, LUMO+1, LUMO+2, and LUMO+3 in the ratio 36:25:21:14 (according to the calculated MOPDOS) and varying ratios of p-wave to s-wave contributions. The yellow circle visualizes the size of a 3D Gaussian broadening with $FWHM = 2.8$ Å applied to the wave function and its lateral derivative, respectively.

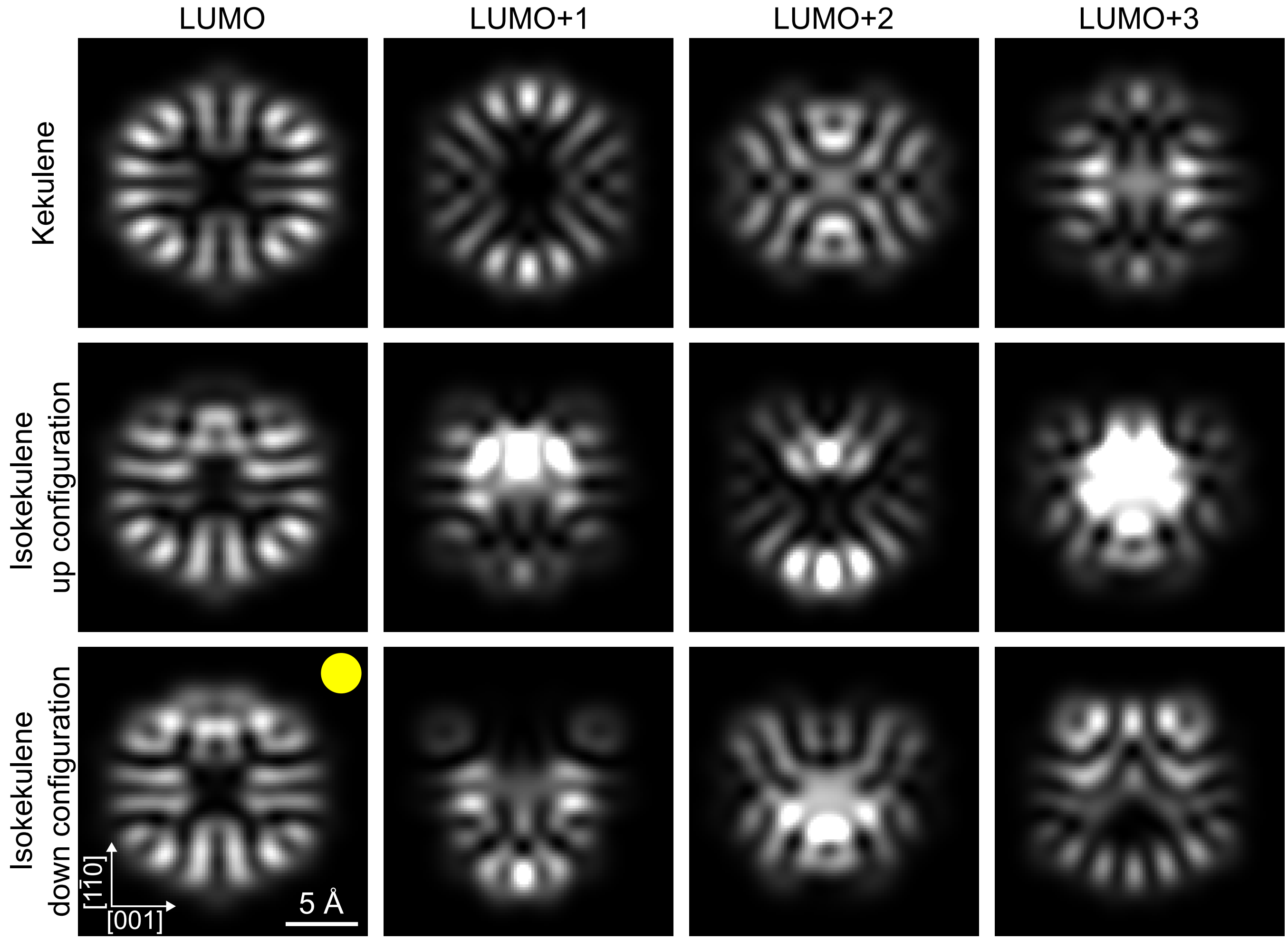

Figure S2: Simulated STM images of kekulene (top row), isokekulene in the up configuration (middle row), and in the down configuration (bottom row) based on single orbitals (from left to right: LUMO, LUMO+1, LUMO+2, and LUMO+3) employing a 3:1 ratio of p-wave to s-wave contributions. For all three species the relaxed geometry of the molecules at the long bride site of Cu(110) has been used. The yellow circle visualizes the size of a 3D Gaussian broadening with $FWHM = 2.8$ Å applied to the wave function and its lateral derivative, respectively.

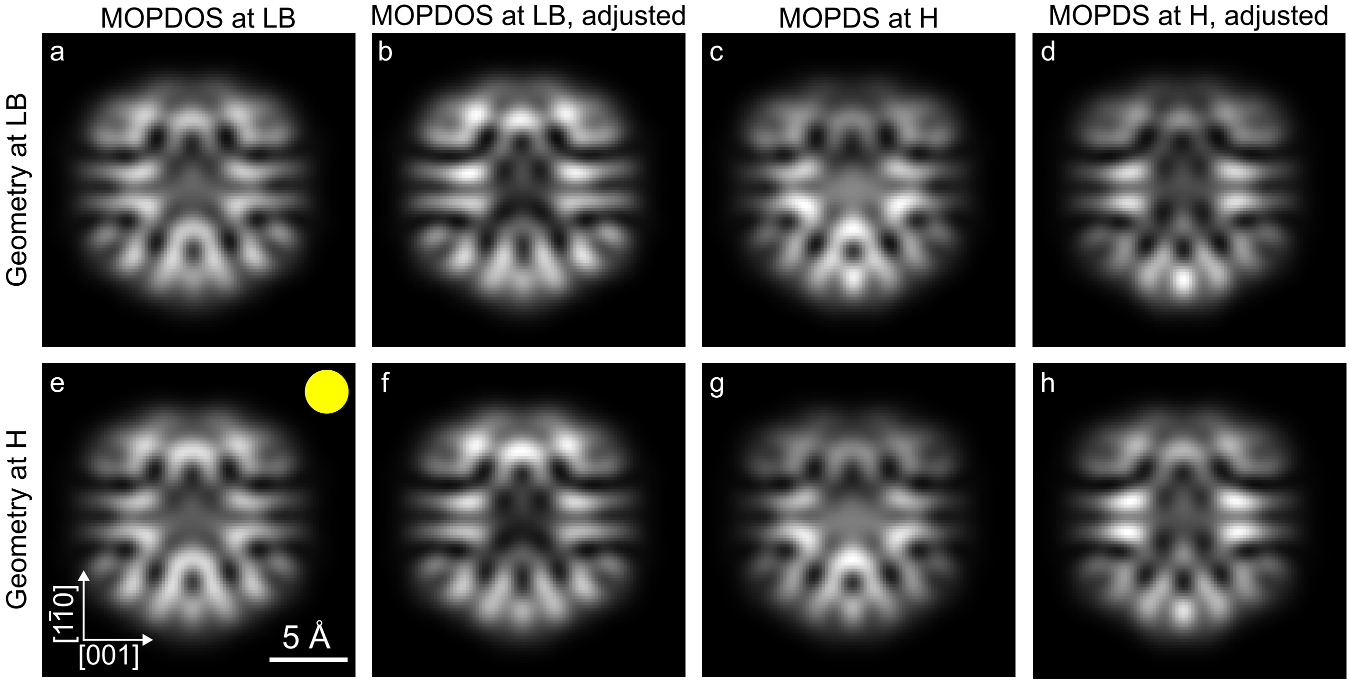

Figure S3: Simulated STM images based on the calculated geometries of isokekulene in the down configuration (top row) at the long bridge (LB) site and (bottom row) at the hollow (H) site on Cu(110) employing a weighted sum of the molecular orbitals LUMO, LUMO+1, LUMO+2, and LUMO+3 in the ratio (first column) 46:8:14:17, (second column) 46:8:0:17, (third column) 23:16:16:10, and (forth column) 23:16:0:10 and a 3:1 ratio of p-wave to s-wave contributions. The yellow circle visualizes the size of a 3D Gaussian broadening with $FWHM = 2.8$ Å applied to the wave function and its lateral derivative, respectively.

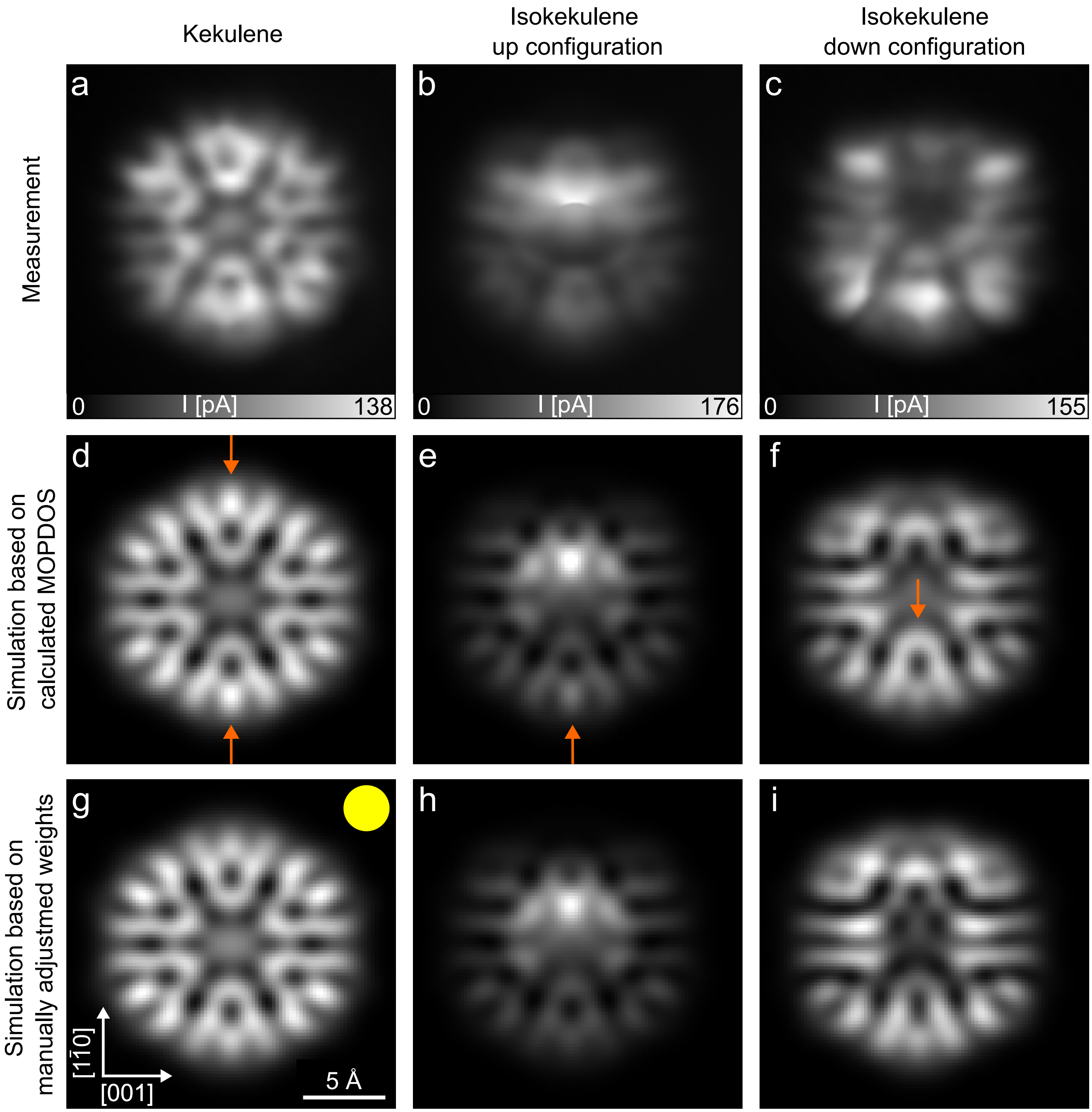

Figure S4: STM images of kekulene (left), isokekulene in the up configuration (middle), and isokekulene in the down configuration (right) on Cu(110), recorded in constant-height mode with a CO-functionalized tip (top row), compared to simulated STM images (middle and bottom row). The simulations employ a p-tip to s-tip ratio of 3:1 and are based on a weighted sum of the molecular orbitals LUMO, LUMO+1, LUMO+2, and LUMO+3 in the ratio (d) 36:25:21:14 (e) 33:23:19:18, and (f) 46:8:14:17 (based on the calculated MOPDOS), as well as (g) 36:13:21:14, (h) 33:23:5:18, and (i) 46:8:0:17 (based on manual adjustments). For all three species the relaxed geometry of the molecules at the long bride site of Cu(110) has been used. The yellow circle visualizes the size of a 3D Gaussian broadening with $FWHM = 2.8$ Å applied to the wave function and its lateral derivative, respectively. Orange arrows mark specific areas which are discussed in detail in the text. The STM images were recorded at 4 K surface temperature after stabilization at (a,c) $U = 5$ mV or (b) $U = 20$ mV and $I = 20$ pA above the copper substrate and subsequently increasing the height of the tip by (a) $z = 120$ pm, (b) 130 pm, and (c) 125 pm.

S2. Supporting Discussion

Figure S4 shows the experimental STM images (Figure S4a-c) and the simulations based on the MOPDOS (Figure S4d-f) as discussed in the main text. In Figure S4g-i, we demonstrate how selective changes to the simulated STM images can be made owing to the flexibility of the presented approach to construct STM simulations as weighted sums of single orbitals. For all three molecules, the brightness in the areas marked with orange arrows is lowered by manually reducing the contribution of one of the orbitals, the LUMO+1 for kekulene and the LUMO+2 for isokekulene in both configurations, in each simulation (see the figure caption for the exact weights), which slightly improves the agreement with the measured images.

The MOPDOS (Figure 3 in the main text) shows that the orbitals in question are strongly broadened due to hybridization. Although the shape of orbitals is a rather robust result of DFT, it is known that the calculated orbital energies can vary depending on the chosen exchange-correlation functional and differ from experimental results in some instances.[1,2] This might be the reason for one orbital possibly being less pronounced for each species here, which examplifies how the detailed comparison to STM measurements might be used to test for possible deviations between theoretical predictions of charge transfer and experimental orbital occupations.

In order to obtain quantitative experimental weights, especially in cases which deviate more significantly from the theoretical predictions, a numerical fit of the experimental data to the simulations of the single orbitals might be appropriate. In comparison to the here presented approach, this would, however, require exact positioning, accurate size-scaling, and precise correction for image distortions of the experimental images. As the agreement between experiment and simulations with the MOPDOS-derived weights is already satisfactory, we have refrained from such a fit in the present case.

S3. Materials and Methods

The Cu(110) single crystal with a miscut of less than 0.1° was purchased from MaTecK GmbH, Germany, and the surface was prepared by cycles of Ar^+ ion bombardment and annealing to 850 K. The precursor was synthesized in solution as reported previously.[3] It was deposited onto the copper surface by evaporation either from a home-built Knudsen cell or from a commercial evaporator from Kentax at 500 K in ultra-high vacuum (UHV).

STM measurements on the low-coverage surface were performed on a commercial Sigma LT instrument at liquid-helium temperatures. For the measurements presented in Figure 1f-h and Figure 4, the commercial qPlus sensor was functionalized by a CO molecule. The measurements on the monolayer were conducted at liquid nitrogen temperature on a SPECS STM Aarhus 150 STM. The images were processed with WSxM[4] and Gwyddion.[5] Background subtraction was applied to the overview STM images (Figure 1a,b).

As described previously,[6] the DFT calculations were performed within a repeated-slab approach employing the Vienna ab-initio simulation package (VASP) and using the projector augmented wave method to treat the core electrons.[7–10] Five layers of the copper substrate are modeled with the theoretical lattice parameter of 3.55 Å as obtained by the PBE-GGA functional including van der Waals corrections according to Tkatchenko and Scheffler.[11] For the adsorption of isokekulene on Cu(110), we use the surface unit cell described by the epitaxial matrix $\left(\begin{smallmatrix} 4 & 3 \\ 0 & 6 \end{smallmatrix}\right)$ as observed by low-energy electron diffraction,[6] while for the adsorption of kekulene the somewhat larger $\left(\begin{smallmatrix} 5 & 0 \\ 0 & 7 \end{smallmatrix}\right)$ overlayer is necessary to properly fit the molecule into the unit cell. For all cases, we consider four adsorptions sites (top, hollow, short bridge, and long bridge with respect to the central void of (iso)kekulene) and perform geometry relaxations including the two topmost layers of the coppers substrate. For the relaxed geometries, we have evaluated the molecular orbital

projected density of states (MOPDOS) and simulated photoemission angular distribution maps (k maps) as described in Ref. 12.

The simulations of the STM images using a fixed ratio of p-wave and s-wave contributions p:s to describe the CO tip are performed following Ref. 13. To this end, we computed the molecular orbitals of the LUMO, LUMO+1, LUMO+2, and LUMO+3 for isolated (iso)kekulene molecules frozen in the adsorption geometry. For each resulting wave function the lateral derivative was calculated and a 3D Gaussian broadening $\mathcal{G}$ was applied in order to approximate the decay of the tip wave functions.[14] After taking the modulus squared, this data was added in a weighted sum over the tip-wave contributions as well as over the molecular-orbital contributions. Overall, the simulated STM current is computed as

$$\sum_i w_i \left(s|\mathcal{G}[\Psi_i]|^2 + p \left(|\mathcal{G}[\partial_x \Psi_i]|^2 + |\mathcal{G}[\partial_y \Psi_i]|^2 \right) \right)$$

from the DFT-calculated wave functions Ψ_i. The orbital weights w_i are taken from the calculated MOPDOS of the adsorbed molecules. Here, the values at the Fermi energy were used and rounded to two-digit values. Finally, a cut at one specific height was taken to obtain 2D constant-height simulations.

ARPES and POT were conducted at the Metrology Light Source insertion device[15] beamline of the Physikalisch-Technische Bundesanstalt (PTB, Germany). The sample was exposed to p-polarized ultraviolet light (35 eV photon energy) with an incidence angle of 40° with respect to the surface normal, and photoelectrons were collected with the toroidal electron spectrometer.[16] For the measurements shown in Figure 2, the photoemission intensity in the emission angle range from 0° (sample normal) to +85° was recorded. Momentum maps were recorded by rotating the sample around its normal in 2° steps and measuring the photoemission intensity at a constant kinetic energy of the electrons.

References

(1) Puschnig, P.; Boese, A. D.; Willenbockel, M.; Meyer, M.; Lüftner, D.; Reinisch, E. M.; Ules, T.; Koller, G.; Soubatch, S.; Ramsey, M. G. et al. Energy Ordering of Molecular Orbitals. *J. Phys. Chem. Lett.* **2017**, *8*, 208–213.

(2) Haags, A.; Yang, X.; Egger, L.; Brandstetter, D.; Kirschner, H.; Bocquet, F. C.; Koller, G.; Gottwald, A.; Richter, M.; Gottfried, J. M. et al. Momentum Space Imaging of σ Orbitals for Chemical Analysis. *Sci. Adv.* **2022**, *8*, eabn0819.

(3) Haags, A.; Reichmann, A.; Fan, Q.; Egger, L.; Kirschner, H.; Naumann, T.; Werner, S.; Vollgraff, T.; Sundermeyer, J.; Eschmann, L. et al. Kekulene: On-Surface Synthesis, Orbital Structure, and Aromatic Stabilization. *ACS Nano* **2020**, *14*, 15766–15775.

(4) Horcas, I.; Fernández, R.; Gómez-Rodríguez, J. M.; Colchero, J.; Gómez-Herrero, J.; Baro, A. M. WSXM: A Software for Scanning Probe Microscopy and a Tool for Nanotechnology. *Rev. Sci. Instrum.* **2007**, *78*, 013705.

(5) Nečas, D.; Klapetek, P. Gwyddion: An Open-Source Software for SPM Data Analysis. *Cent. Eur. J. Phys.* **2012**, *10*, 181–188.

(6) Ruan, Z.; Fan, Q.; Reichmann, A.; Kang, F.; Naumann, T.; Werner, S.; Kleykamp, O.; Martinez-Castro, J.; Lüpke, F.; Haags, A. et al. Highly Structure-Selective On-Surface Synthesis of Isokekulene Versus Kekulene. *Angew. Chem., Int. Ed.* e202509932.

(7) Kresse, G.; Hafner, J. Ab Initio Molecular Dynamics for Liquid Metals. *Phys. Rev. B* **1993**, *47*, 558–561.

(8) Kresse, G.; Furthmüller, J. Efficiency of Ab-Initio Total Energy Calculations for Metals and Semiconductors Using a

Plane-Wave Basis Set. *Comput. Mater. Sci.* **1996**, *6*, 15–50.

(9) Kresse, G.; Furthmüller, J. Efficient Iterative Schemes for Ab Initio Total-Energy Calculations Using a Plane-Wave Basis Set. *Phys. Rev. B* **1996**, *54*, 11169–11186.

(10) Kresse, G.; Joubert, D. From Ultrasoft Pseudopotentials to the Projector Augmented-Wave Method. *Phys. Rev. B* **1999**, *59*, 1758–1775.

(11) Tkatchenko, A.; Scheffler, M. Accurate Molecular Van Der Waals Interactions from Ground-State Electron Density and Free-Atom Reference Data. *Phys. Rev. Lett.* **2009**, *102*, 073005.

(12) Lüftner, D.; Weiß, S.; Yang, X.; Hurdax, P.; Feyer, V.; Gottwald, A.; Koller, G.; Soubatch, S.; Puschnig, P.; Ramsey, M. G. et al. Understanding the Photoemission Distribution of Strongly Interacting Two-Dimensional Overlayers. *Phys. Rev. B* **2017**, *96*, 125402.

(13) Gross, L.; Moll, N.; Mohn, F.; Curioni, A.; Meyer, G.; Hanke, F.; Persson, M. High-Resolution Molecular Orbital Imaging Using a p -Wave STM Tip. *Phys. Rev. Lett.* **2011**, *107*, 086101.

(14) Paschke, F.; Lieske, L.-A.; Albrecht, F.; Chen, C. J.; Repp, J.; Gross, L. Distance and Voltage Dependence of Orbital Density Imaging Using a CO-Functionalized Tip in Scanning Tunneling Microscopy. *ACS Nano* **2025**, *19*, 2641–2650.

(15) Gottwald, A.; Kaser, H.; Kolbe, M. The U125 Insertion Device Beamline at the Metrology Light Source. *J. Synchrotron Radiat.* **2019**, *26*, 535–542.

(16) Broekman, L.; Tadich, A.; Huwald, E.; Riley, J.; Leckey, R.; Seyller, T.; Emtsev, K.; Ley, L. First Results from a Second Generation Toroidal Electron Spectrometer. *J. Electron Spectrosc. Relat. Phenom.* **2005**, *144–147*, 1001–1004.
