# Supplementary material for: Multi-Orbital Charge Transfer into Nonplanar Cycloarenes Revealed with CO-Functionalized STM Tips
Source: J Phys Chem Lett. 2026 Jan 21;17(5):1296–304. doi: 10.1021/acs.jpclett.5c03268 (PMC12884524; doi:10.1021/acs.jpclett.5c03268)
Supplement: Supplementary file 2 [file jz5c03268_si_002.pdf]

Peer Review Information for

“Multi-Orbital Charge Transfer into Nonplanar Cycloarenes Revealed with CO-Functionalized STM Tips”

jz-2025-03268c.R1

First Round of Reviewer Comments

Author’s Response to the Reviewer Comments

Reviewer: 1

Recommendation: This paper represents a significant new contribution and should be published as is.

Comments:

Haags et al investigated the interfacial electronic structure of kekulene and isokekulene on copper surfaces. The selective synthesis of kekulene on Cu(111) and isokekulene on Cu(110), reported recently by some of the authors (Ref. 18), enables the investigation of these molecules using also laterally averaging experimental methods. The manuscript greatly benefits from the combination of laterally integrating angle-resolved photoemission (more precisely: photoemission orbital tomography, POT) and scanning tunneling microscopy (STM), supported by extensive DFT calculations. This approach might be of high importance for future investigations of related systems, even if minor discrepancies between constant-height STM images and simulations based on DFT remain. It is clearly demonstrated that a charge transfer into multiple formerly unoccupied molecular orbitals of kekulene and isokekulene occur at the interface to copper. The manuscript is well written and of very high scientific interest. “The Journal of Physical Chemistry Letters” might be an appropriate journal for this work. I recommend publishing the manuscript as is.

We thank the reviewer for their assessment of our manuscript and the positive recommendation.

Minor remark: Is it possible to compare the simulated molecular orbital projected density of states (MOPDOS) in Figure 3(f-j) to experimental (integrated) intensity-energy spectra?

We thank the reviewer for their question. In principle, only the total density of states (meaning the sum over the contributions from the different orbitals) could be measured by scanning tunneling spectroscopy. However, the spectra we recorded did not show a clearly defined gap or sharp, distinct peaks in comparison to the spectrum of the bare surface. This is likely due to the strong hybridization between surface and molecular states and prevents a comparison of the calculated total DOS to a measurement in our case. The comparison of the MOPDOS to a measurement via the simulated STM images, as presented in the manuscript, offers more detailed insights as the occupation of the single orbitals can be differentiated.

Additional Questions:

Urgency: Top 10%

Significance: Top 10%

Novelty: Top 10%

Scholarly Presentation: Top 10%

Is the paper likely to interest a substantial number of physical chemists, not just specialists working in the authors' area of research?: Yes

Reviewer: 2

Recommendation: This paper may be publishable, but major revision is needed; I would like to be invited to review any future revision.

Comments:

This paper investigates partial charge transfer from the surface, Cu(111) and Cu(11), into several formerly unoccupied molecular orbitals of single isolated adsorbed molecules, kekulene and isokekulene in different adsorption conformations. It is based on STM and photoemission orbital tomography measurements and theory. Main findings result from simulating STM images based on a weighted sum of different molecular orbitals, employing weights based on the calculated molecular-orbital projected density of states and fitting them to STM maps. The weights are the fitting parameters. That way, partial occupations of the former LUMO orbitals are extracted.

The data is of very high quality, and the findings are interesting. The quantification of partial charge transfer of single molecules on metals has important implications and is difficult to access. I support publication, after my comments have been addressed.

We thank the reviewer for their assessment and detailed comments. As described point by point below they have allowed us to significantly improve our work.

1. Can the total amount of partial charge transfer be estimated using this method? This would be a very important result of the paper, but it is not discussed. In relation to this question: Do the former HOMO orbitals remain fully occupied, or is there back donation? A discussion of these points would be nice.

The statement on p. 6 "A similar degree of charge transfer and hybridization as for kekulene on Cu(110) is also found for isokekulene on Cu(110)...", hints that overall charge transfer is accessed with the technique. But the point about quantification is not elaborated.

We thank the reviewer for raising this important point. In brief, the total amount of charge transferred between the molecule and the substrate cannot be quantitatively determined using our method. The approach we use allows, in principle, only the extraction of partial

occupation numbers of the formerly unoccupied molecular orbitals (LUMO, LUMO+1, …). However, summing up these partial occupations does not yield the net charge transfer from the metal to the molecule.

The reason is that adsorption leads to hybridization of all molecular states with the substrate continuum, which in turn causes a redistribution of electron density that also involves the formerly fully occupied orbitals (HOMO, HOMO–1, …). In other words, back-donation from the molecule to the substrate necessarily accompanies electron donation into the LUMO-derived states. This redistribution implies that the apparent population of the LUMO manifold is only one part of the overall charge-transfer process.

More generally, we would like to emphasize that the total charge on a molecular fragment in an adsorbate-substrate complex is, strictly speaking, not a uniquely defined observable, because there is no uniquely defined way to partition the electron density of the combined system into a "molecular" and a "substrate" contribution. Different charge-partitioning schemes can yield different numerical values. In earlier work [R1], we have employed a Bader charge analysis as a representative density-based partitioning method. These calculations revealed that the net charge transferred to the molecule can be significantly smaller than the sum of the partial occupations of the formerly unoccupied orbitals. This further illustrates that LUMO occupation alone does not provide a quantitative measure of the total charge transfer.

For clarity, we have included how the MOPDOS is calculated (main text page 6, lines 31-33), adjusted the wording to "partial charge transfer" (page 6, line 67), and added a short discussion on the hybridization of the HOMOs (main text page 6, lines 72-81).

[R1] T. Boné *et al.*, "Demonstrating the Impact of the Adsorbate Orientation on the Charge Transfer at Organic-Metal Interfaces", *J. Phys. Chem. C* 125, 9129-9137 (2021) https://doi.org/10.1021/acs.jpcc.1c01306

2. Please provide colour scales for the STM measurements. That is, heights in Fig. 1 and, importantly, currents in Fig. 4.

We thank the reviewer for the comment and have added color bars to all measured STM images in Figure 1 and Figure 4. In order to ensure that a correct color bar can be given, we have newly exported the image in Figure 1b from the raw data resulting in a slightly different appearance of the colors in the revised figure. This also led to a statement about image filters in the Methods section being revised.

3. The simulation and fitting of the orbital density maps are essential to the paper. There might be an issue. But I do not know how important it is.

a. Please provide more details in the SI about how the images were simulated and fitted to the experiment.

We thank the reviewer for the suggestion and agree that a more detailed description of the method presented in the manuscript is useful. We wish to especially clarify that the weights

of the different orbitals are not the result of a numerical fit to the experiment, but stem from the calculated MOPDOS. In this way, a fairly simple calculation of a weighted sum of the simulated tunneling currents for the single orbitals is sufficient (and no fitting is required) to achieve an overall simulation, which can be compared to the measurement in order to confirm the occupation of the former LUMOS as predicted by the DFT. The only deviation from this is that in a second step (now Figure S4g-i) the contribution of one orbital is reduced manually to test for possible deviations between theory and experiment.

For clarity, we have included more details about the STM simulation as well as about the calculation of the MOPDOS (see also the answer to question 1) in the main text (page 8, lines 69-86) and in the Methods section (SI page 7) including an equation clearly showing the order of the calculations (see also the following question). We have included the reviewer's idea of a numerical fit in the supporting discussion (SI page 6).

b. Gaussian broadening was applied to the simulated STM images. (Figure S1). It seems that the broadening was applied after (?) simulating the p-wave tip imaging (although it is not explicitly explained how it was done). Caption Fig. S1: "Gaussian broadening was applied to the simulated STM images, which corresponds to a tip radius of 0.4 Å." Implies it is done after simulating the p-wave contrast. This would be questionable. I think the broadening should be applied to the derivatives of the orbital wavefunction before taking the absolute squares to obtain the density image. Details can be found in F. Paschke et al, "Distance and Voltage Dependence of Orbital Density Imaging Using a CO-Functionalized Tip in Scanning Tunneling Microscopy," ACS nano, vol. 19, pp. 2641–2650, 2025. (see in particular Fig. S10).

Let me quickly explain on an exemplary point: above a (s-wave like) wavefunction maxima, a p-wave tip should result in zero current – even for large distances (large blurring). If the p-wave image is first calculated and then broadening is applied, this will result in currents at this tip position. Thus, the procedure of applying broadening in the end (after taking the abs. square) seems to produce features that are similar to those of a s-wave tip contribution.

We thank the reviewer for their detailed explanation. Indeed, the 3D Gaussian broadening had originally been applied after the modulus squared. As the broadening value had been chosen small, the results were qualitatively similar to the same images completely without broadening (see the answer to question 3c and Figure R1). However, implementing the broadening before the modulus squared following Paschke *et al.*, as kindly suggested by the reviewer, allowed us to significantly improve the agreement between our simulation and the measurements. Whereas for small broadening (up to around $\sigma = 0.4$ Å) we obtain similar results as with the previous method, the correct broadening changes the appearance more significantly when moving to larger values. The best agreement between the simulations and the measurements is found at $\sigma = 1.2$ Å (or $FWHM = 2\sqrt{2\ln 2}\sigma = 2.8$ Å) (especially when choosing a p to s ratio of 3:1, see the answer to the following question). This choice was made for the revised Figure 4, which now focuses only on the exact weights from the calculated MOPDOS, which are well confirmed. A refined discussion of the possibility of manual adjustments to test for possible (but in this case rather small) discrepancies between calculated and experimental

orbital occupations has been moved to the supporting information (page 6) and is referred to in the main text on page 10, lines 10-15.

Additionally, the discussion of the remaining discrepancy for isokekulene in the down configuration could be focused more precisely on specific areas of the molecule and the revised conclusion that DFT underestimates the distortion compared to the experimental geometry is now more reliable (page 10, lines 18-33 and lines 53-65).

We have adjusted the explanation of the broadening in the main text (page 8, lines 74-78), Methods section (page 7 of the SI) and revised the captions to Figures 4, S1, S2, S3, and S4 accordingly. In order to visualize the size of the Gaussian broadening, we have added circles with the *FWHM* as the diameter to the figures. As opposed to reporting the σ value of the Gauss as in the original manuscript, we have chosen to report the *FWHM* value in the revised manuscript as it is clearer to visualize.

c. Related to the former point: The statement: "Both s-wave and p-wave contributions were considered in the simulation, but any inclusion of s-wave contributions reduced the agreement with the experiment." should be reconsidered. Anyway, the statement is hard to believe, as the addition of s-wave contribution provides more fitting parameters and thus should likely improve the fit. If the authors would state that they only use p-wave tip contributions, because they assume these are dominant wrt. s-wave contributions, and provide an argument for that, that would be acceptable, I think. But if they make the statement above, they should corroborate it, e.g. fitting with allowing both s- and p-wave tip contributions and then finding vanishing contributions of the s-wave tip. Related to the previous point: I assume that s-wave tip does contribute, and that the fit with p-wave tip improves, because the broadening is applied in the end.

Note that the correct simulation of STM images is essential to the paper, as changing it will affect the extracted contributions of partial orbital occupations.

Having said this, I assume that the main findings of the paper will not change much as the applied broadening was small, with a small tip radius of only 0.4 Å. An option to avoid the issue might be to not apply any broadening (instead of a "wrong broadening").

If the authors think that how they apply the broadening is justified, they should state very clearly how they applied it, and possibly provide arguments for it.

We thank the reviewer for their comment. As no numeric fit was applied (see answer to part a. of the question), a potential contribution of *s*-wave tunneling was tested manually by including it in varying amounts and observing the agreement with the measurement. After implementing the correct broadening (see previous point) we could re-evaluate the choice of *p* to *s* ratio. To clarify this process, the additional Figure S1 was added to the SI, which shows the same simulation of kekulene with varying *p* and *s* contributions. In comparison to the measured image, we have chosen a 3:1 ratio for all other simulations presented in the main text (revised Figure 4) as well as the SI (revised Figures S2-S4).

We wish to clarify that although we aim to confirm the orbital weights by comparing to the measurements, the given occupations are based solely on the calculated MOPDOS (Figure 4d-f) and the manual adjustment of reducing the contribution of one orbital (Figure S4g-i). Thus, the extracted contributions are confirmed via the simulations but not a direct result of the STM simulations themselves. Generally, the improved agreement between measured and simulated images further supports the general conclusions regarding the partial charge transfer into the former LUMOs, which remain unchanged.

We thank the reviewer for their idea to test the simulations without Gaussian broadening. As can be seen below for the three different molecules (with the orbitals' weights based on the MOPDOS as in the middle row of Figure 4d-f), this is qualitatively comparable to the simulations in the original manuscript (as the width of the broadening was small). However, the differences in comparison to the revised Figure 4 and the revised Figure S1 suggest that the "wrong" broadening might have indeed led to some artificial s-wave contributions. As described above, the correct broadening (before the modulus squared) has improved the agreement with the measurement significantly, such that this method was chosen in combination with the *p* to *s* ratio of 3:1 in the revised manuscript.

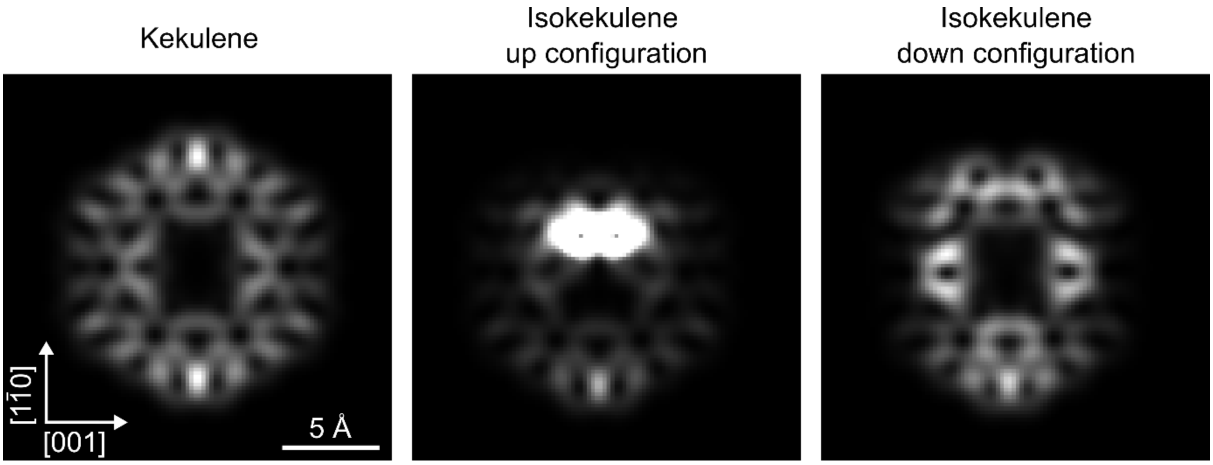

Figure R1: p-wave simulations of the three molecules without Gaussian broadening employing the weights from the MOPDOS (as in Figure 4d-f in the manuscript).

Additional Questions:

Urgency: Top 10%

Significance: Top 10%

Novelty: Top 10%

Scholarly Presentation: Moderate

Is the paper likely to interest a substantial number of physical chemists, not just specialists working in the authors' area of research?: Yes

jz-2025-03268c.R2

Second Round of Reviewer Comments

Reviewer: 1

Comments to the Author

The response of the authors is conclusive. The manuscript might be published as is.

Reviewer: 2

Comments to the Author

The authors did an excellent job in revising and improving their paper. I recommend publication as is.
